# Analysis of diesel engine injector nozzle spray characteristics fueled with residual fuel oil

**DOI:** 10.1016/j.heliyon.2020.e04637

**Published:** 2020-08-07

**Authors:** C.H. Achebe, B.M.O. Ogunedo, J.L. Chukwuneke, N.B. Anosike

**Affiliations:** a Department of Mechanical Engineering, Nnamdi Azikiwe University, Awka, Nigeria; b Department of Mechanical Engineering, Imo State University, Owerri, Nigeria

**Keywords:** Chemical engineering, Energy, Industrial engineering, Mechanical engineering, Constant volume spray chamber, Diesel engine, Injection system, Residual fuel oil, Rich mixture, Sauter mean diameter, Spray cone angle, Spray length, Spray volume

## Abstract

Experimental analysis on the spray characteristics of a diesel engine injector nozzle fueled with Residual Fuel Oil (RFO) was carried out in this study. To achieve this, the fuel was characterized to determine its physicochemical properties, and an experimental set up was designed to visualize and capture the spray pattern of the fuel. The images obtained were processed and analysed using Image J software to determine the spray length, spray cone angle, spray area, spray volume, and spray velocity values of the fuel. Experimental results obtained agree with validation models and reveal that spray parameter values of RFO are higher than those of diesel fuel. The values of spray parameters of RFO such as 456mm spray length, 2.85mm Sauter Mean Diameter (SMD) and the low spray cone angle of 12.69°, led to a higher spray volume causing the engine to run on a rich mixture after initial start-up conditions. This would create such challenges as reduction in power and clogging of injector nozzle tip due to an increase in carbon deposits. Regression models generated reveal that these challenges could be eliminated when the spray parameters run on optimal values of 256mm, 6.41cm^2^, 16.18cm^3^, 0.96 mm/s and 13.59° for the spray length, spray area, spray volume, spray velocity and the spray angle respectively. These optimal values were obtained when the engine fuel injection time was set to 500μs while running on fuel of viscosity 4.305 mPa.s and temperature of 48 °C.

## 1 Introduction

Internal combustion engine technology has diesel engines traditionally preferred in heavy-duty applications for their fuel economy, robustness, and reliability. For instance, in Nigeria today, the electric power challenge faced by the country has forced manufacturing/production companies to rely mostly on internal combustion power plants as prime movers for the production of electricity. These power plants include petrol, diesel, and gas engines, but diesel and gas engines favour heavy duty and high energy demand application with diesel engines having advantages of lower running and capital costs, ease of installation, simple plant layout, and availability of fuel over the gas engines and hence, are widely used in the manufacturing/production industries [1, 2]. The Fuel injection systems in modern diesel engines are designed to meet the highest level of fuel economy, drivability, and rapid response, easy and accurate control while complying with very stringent environmental standards. Changing fuel characteristics could affect the performance of the fuel injection system.

Some researches [3, 4, 5, 6] have been carried out on combustion systems in diesel engines to determine the phenomena that occur when an active fluid (fuel) is injected into a working fluid (air) under standard thermodynamic conditions. With the advancement in technology, it is possible to characterise the injection fuel process in the combustion chamber under experimental conditions that match those happening when the engine is running under standard conditions. The injection process is examined based on the interaction between the fuel injected into the combustion chamber and the air. In doing this, the fuel spray structure becomes a strong basis for analysis. Hence, it is important to obtain maximum control of the fuel spray structure using electronic control systems in the experiments for cheaper surrogate fuels that could reduce pollutant emissions while achieving a good engine performance. This will enhance the knowledge of the spray parameters that would influence the desired outcome, since the spray parameters have a direct influence on combustion, which in turn affects the engine performance and emission characteristics. With the steady decline in power generation and distribution in the country, manufacturing and production outfits, especially the small and medium enterprises (SMEs) are forced to rely heavily on stand-alone diesel engine generating sets to meet their electric power needs. This adds to their cost of production and could cause loss of market influence in a competitive market.

Hence, the need to cut production costs through reduction in the cost of fuelling the power generating sets. This thus calls for the use of cheaper surrogate (unconventional) fuel in the face of soaring and expensive oil price as an imperative. However, this comes with heavy penalties such as deposit formation in nozzle tip, inability of the generating set to re-start after shutdown, and subsequent degradation/failure of the injector nozzle. Researches carried out on the relationship between the physical properties of liquid fuels and spray characteristics [7, 8, 9, 10, 11] reveal that the physical properties of a liquid fuel are the intrinsic factors affecting its spray characteristics. Therefore it is safe to say that the microstructure and physical properties of unconventional fuels differ from conventional diesel fuel in key characteristics such as spray volume, spray cone angle, spray morphology, spray penetration, and Sauter Mean Diameter (SMD). These characteristics have been discovered to be related to the quality of fuel-air mixing under varying pressure conditions. It is then only expected that conventional diesel fuel and unconventional fuels will yield different results in the quality of mixing as a result of differences in physical properties.

To this end, this research is in response to a current challenge encountered by manufacturing companies in Anambra State of Nigeria which try to use Residual Fuel Oil (RFO) as a surrogate fuel to achieve lower cost of production. This practice has however necessitated deposit formation in the injector nozzles impeding the flow of fuel and causing inability of the engines to start after being shut down.

## 2 Materials and methods

**Figure 1:**
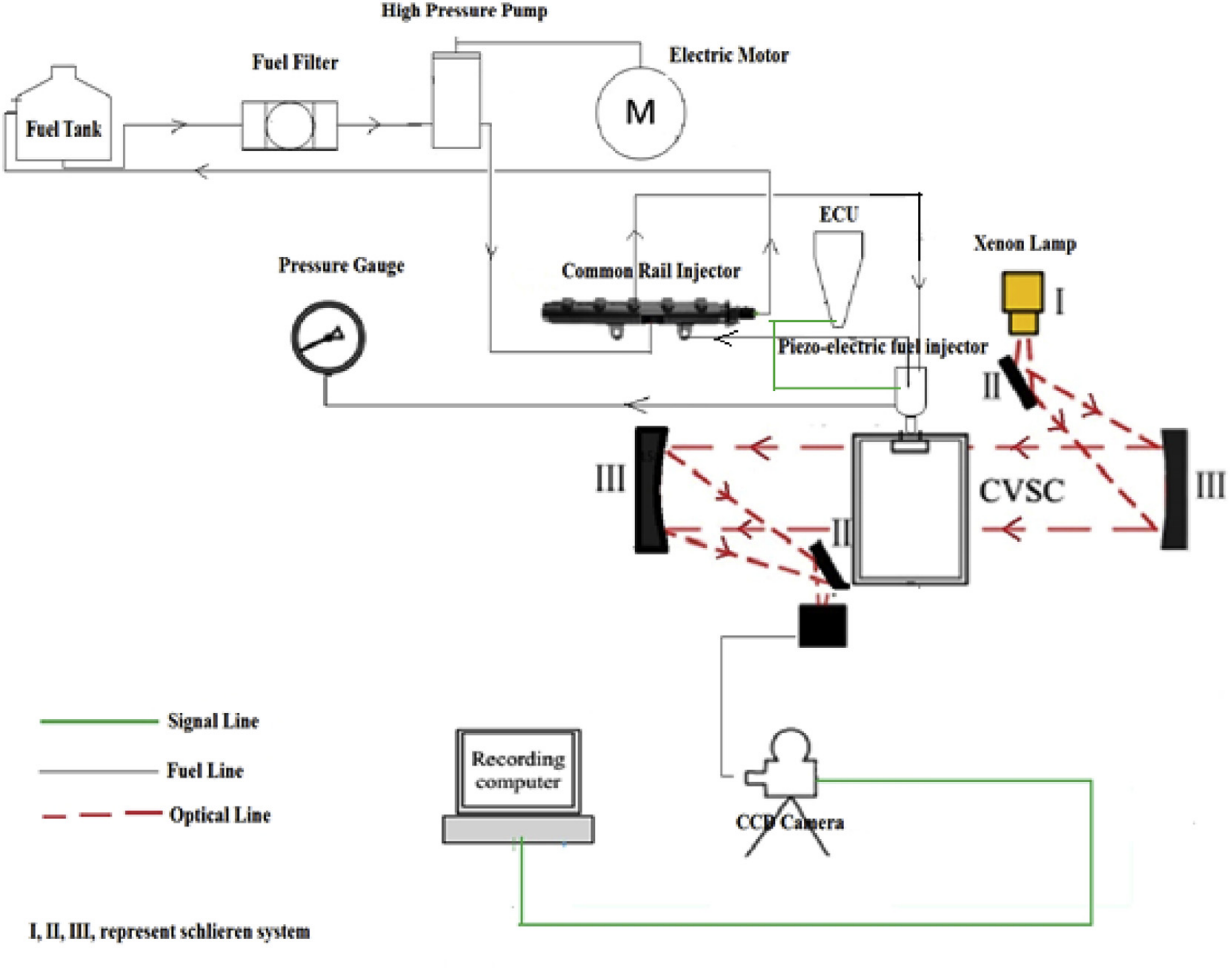
Experimental layout for schlieren visualization technique.

The experimental set up is shown in Figure 1. The set up consists of a visualization and image processing system, constant volume spray chamber (CVSC), fuel supply and injection system, and a diesel injector tester unit.

### 2.1 Visualization and imaging system

The method of spray observation adopted in this study is the Schlieren visualization technique [12]. The Schlieren apparatus enables the spray pattern within the CVSC to be monitored and recorded for onward image processing. It consists of an illumination source (Xenon lamp), a pair of a convex lens with a focal length of 30cm, a knife-edge for cutting off blurry image effect, and a Photron Fastcam SA5 high-speed camera. The camera sensor which is a CCD type is 12bit monochrome with a spatial resolution of 20mm pixel with a minimum exposure time of 1ms. The images were captured at 20,000fps with a maximum spatial resolution of 832 by 448pixels and temporal resolution of 0.05ms. The spray image obtained was processed using Adobe Photoshop and the spray characteristics were quantified using ImageJ code.

### 2.2 Constant volume spray chamber (CVSC)

The CVSC is the environment where the spray is carried out. It is made up of BS1449–S1.2 (1991) steel and has a volume of 4litres. To allow for visualization, a 90mm diameter hole is cut through the CVSC and a Quartz glass of 5mm thickness is fixed.

### 2.3 Fuel supply and injection system

The system is made up of the following: a 4litre fuel tank; an electric motor of 1.5hp, 59.7Nm, and 300rpm; a 1hp oil pump; a flexible fuel hose; a high-pressure fuel delivery pipe and a Perkins 2800 model injector. These components function to ensure that the fuel gets to the injector for subsequent spray action.

### 2.4 Diesel injector tester

The unit is a CR-C common rail diesel injector tester. It is connected to the injector so it can monitor fuel injection condition. It is designed to operate on four drive modes viz: Bosch, for Bosch brand injectors; Denso, for Denso brand injectors; Delphi, for Delphi brand injectors, and General, for other brands of injectors. In controlling the fuel injection conditions, it performs a frequency and pulse parameter adjustment function. The frequency parameter adjustment controls the number of times the injector needle will lift within a given period, in other words, it determines the number of sprays while the pulse controls the duration of spray.

### 2.5 Experiment parameters

The experiment was designed to visualize the spray pattern of Residual Fuel Oil (RFO) to determine the macroscopic and microscopic spray parameters such as spray penetration depth, spray cone angle, spray volume, spray area, spray velocity, and Sauters Mean Diameter (SMD). The sample population for the Residual Fuel Oil (RFO) is the fuel used in a manufacturing industry located at Awka II industrial layout. A sample unit of four litres from the sample population was used in experimenting. The sample unit was characterized to determine the physical properties of the RFO, which researches carried out by [6, 7, 8, 9, 10] opine that they affect the spray parameters of the fuel. These physical properties and the time of injection (ToI) make up the primary variables of the experiment since they are independent variables that are possible sources of variation in the expected outcome of the experiment. The background variables of the experiment are the spray parameters of interest such as spray penetration depth, spray cone angle, spray volume, spray area, spray tip velocity, and Sauters Mean Diameter (SMD), while factors such as pressure and the injector nozzle diameter are the constant variables in the experimental design. The experiment seeks to replicate a compression ignition (CI) fuel spray pattern using an existing piezoelectric fuel injector obtainable in the diesel engines used in the manufacturing industry. There were a total of 42 runs, 7 runs, 6 runs, 6 runs and 42 runs for the determination of the spray tip penetration length, spray cone angle, spray area, spray volume, and spray tip velocity respectively. An objective function will then be developed from the experimental outcome in order to determine optimal parameter values for running the engine on RFO.

### 2.6 Mode of experiment

**Figure 2:**
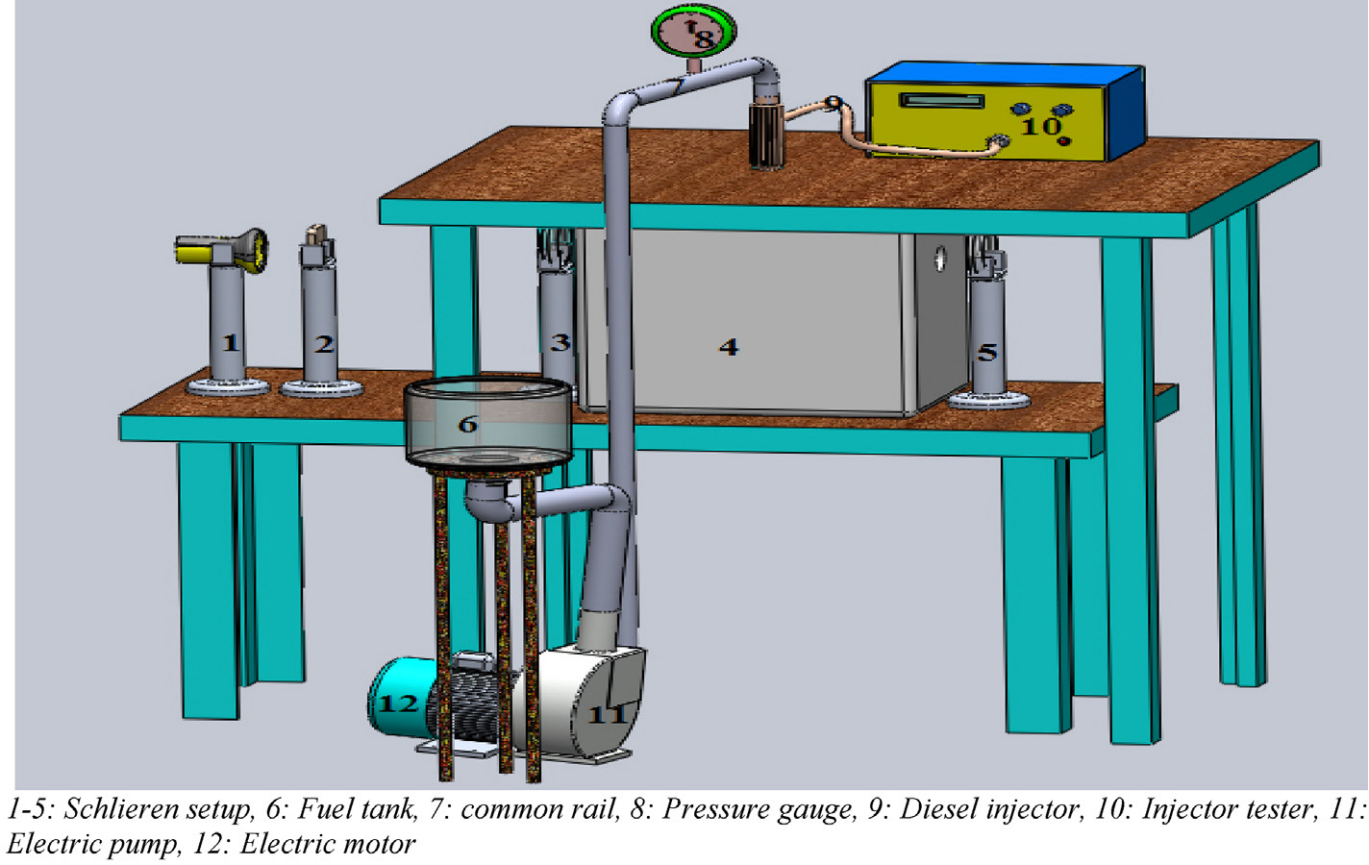
3D model of the Experimental Layout for Schlieren Visualization Technique.

The sample unit (fuel) was collected from an industry running on RFO after it had gone through the processes of decantation, heating and filtration. With the aid of an electric motor, the high-pressure pump was energized to provide and maintain the high injection pressure needed by the common rail injector. An Injector tester unit was installed between the common rail injector and the piezoelectric fuel injector to determine the amount of fuel to inject into the CVSC per time. A visualization opening of 90mm was made in the CVSC and covered with quartz. As light from the Xenon lamp passes through the lens and falls on the spray, the changes in density between the air and the spray in the CVSC experienced by the light rays cause a change in the local refractive index. This refracted image produced was captured by the CCD camera and recorded using the computer system. The images recorded were processed and used to determine the spray parameters under investigation. Figure 1 gives the schematics of the experimental setup. Figure 2 gives a computer-aided 3D model of the experimental setup.

### 2.7 Validation models

Although the schlieren visualization technique gives clear images for the observation of macroscopic spray parameters, it is quite limited in capturing images for microscopic spray parameter visualization. Hence, to overcome this limitation the following models were used to validate the macroscopic parameters while determining the microscopic spray parameters. The one-way analysis of variance (ANOVA) method was used in the analysis for comparison.

#### 2.7.1 Spray penetration length

This is the maximum distance travelled by the spray before making contact with the walls of the combustion chamber. The spray penetration length affects the combustion quality of the fuel; shorter spray lengths produce better combustion than longer spray lengths. The validation model for the penetration length is derived based on the momentum conservation and continuous jet theory [13]. The continuous jet theory suggests that the spray length is directly proportional to the square root of the spray time. Mathematically, this is expressed as

(1) $$S\alpha \sqrt{t}$$

At breakup length, t = t_b_, therfore;

(2) $$S=\beta \sqrt{t}$$

(3) $$S=\beta \sqrt{t_{b}}$$

Also, injection velocity at the break up length, V_inj_ is expressed as: $V_{inj}=\frac{l_{b}}{t_{b}}$

Therefore;

(4) $$S\left(t\right)=\sqrt{C_{d}D_{n}t}{\left[\frac{2\text{Δ}P_{inj}}{\rho_{a}}\right]}^{0.25}$$

**Figure 3:**
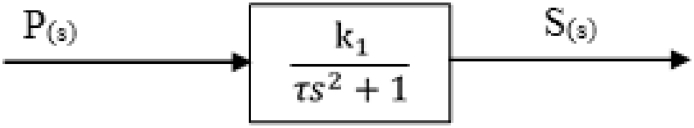
Transfer function block for spray tip penetration length.

The transfer function block was developed from Eq. (4) and was used to generate code blocks in MATLAB which was run in Simulink to validate the spray tip penetration result. The transfer function block is expressed in Figure 3.

**Figure 4:**
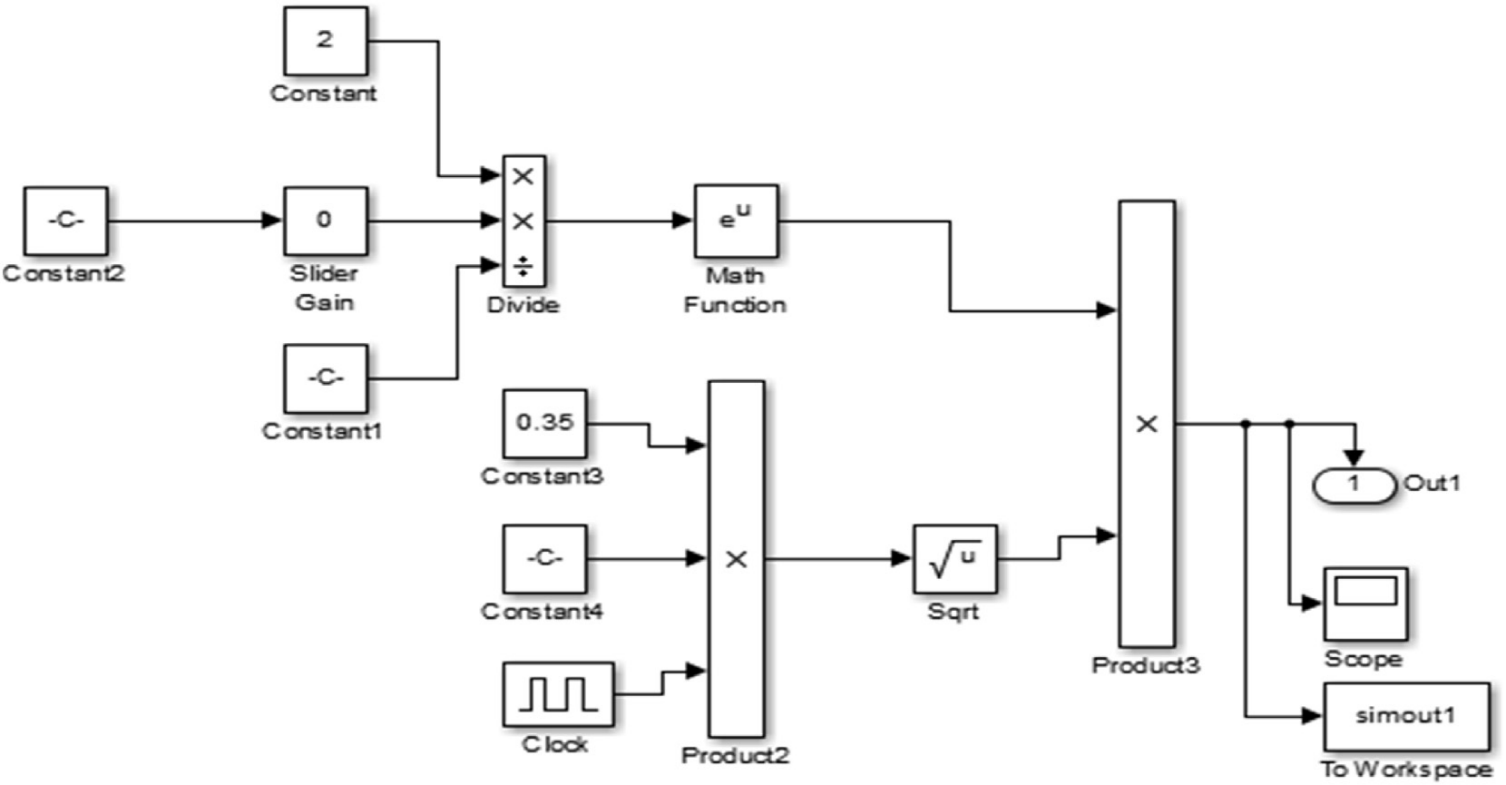
Spray tip penetration length code block.

Figure 4 gives the code blocks for the determination of the spray tip penetration.

#### 2.7.2 Spray tip velocity

This is the speed at which the fuel travels within the spray chamber. The spray tip velocity is determined using the relation [14].

(5) $$U_{s}\left(t\right)=\frac{2.95}{2}{\left(\frac{\text{Δ}P_{inj}}{P_{f}}\right)}^{0.25}{\left(\frac{d}{t}\right)}^{0.5}=\frac{S\left(t\right)}{t}$$

**Figure 5:**
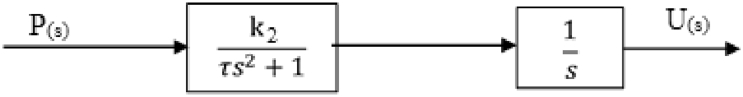
Transfer function block for spray tip velocity.

The transfer function block was developed from Eq. (5) and was used to generate code blocks in MATLAB which was run in Simulink to validate the spray tip penetration result. The transfer function block is expressed in Figure 5.

**Figure 6:**
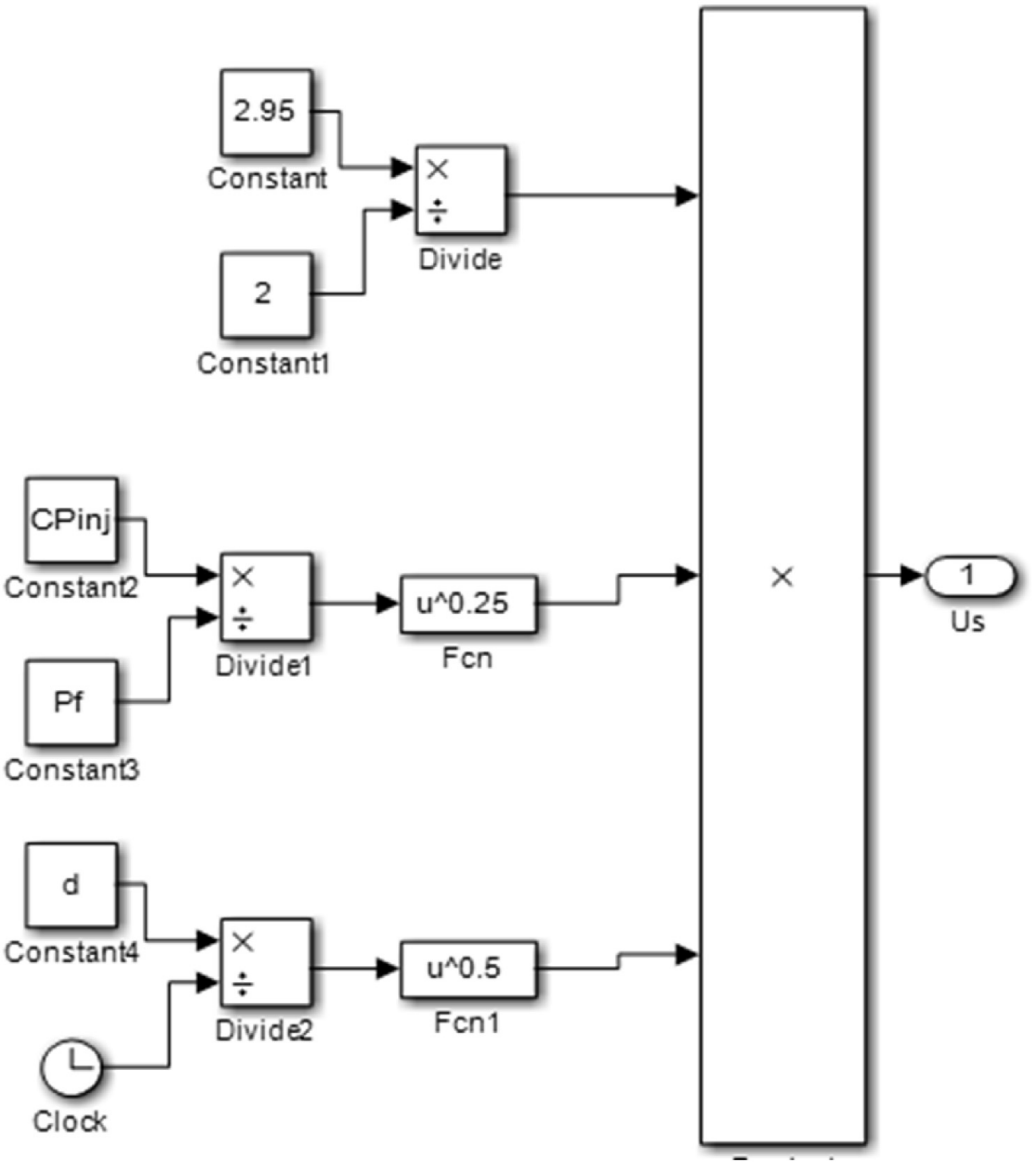
Spray tip velocity code block.

Figure 6 gives the code blocks for the determination of the spray tip velocity.

#### 2.7.3 Spray cone angle

**Figure 7:**
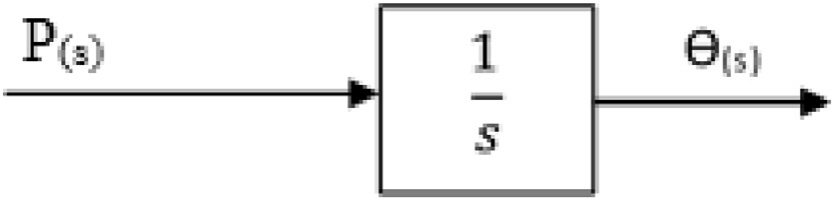
Transfer function block for spray cone angle.

This is the largest angle formed by two straight lines at the spray boundary in the combustion chamber. The spray cone affects the penetration length. Increase in the spray cone reduces the penetration length. The cone angle was determined from the processed image of the spray and result from the equation used by [15]. The transfer function block as shown in Figure 7 was developed and used to generate code blocks in MATLAB which was run in Simulink to validate the experimental result.

**Figure 8:**
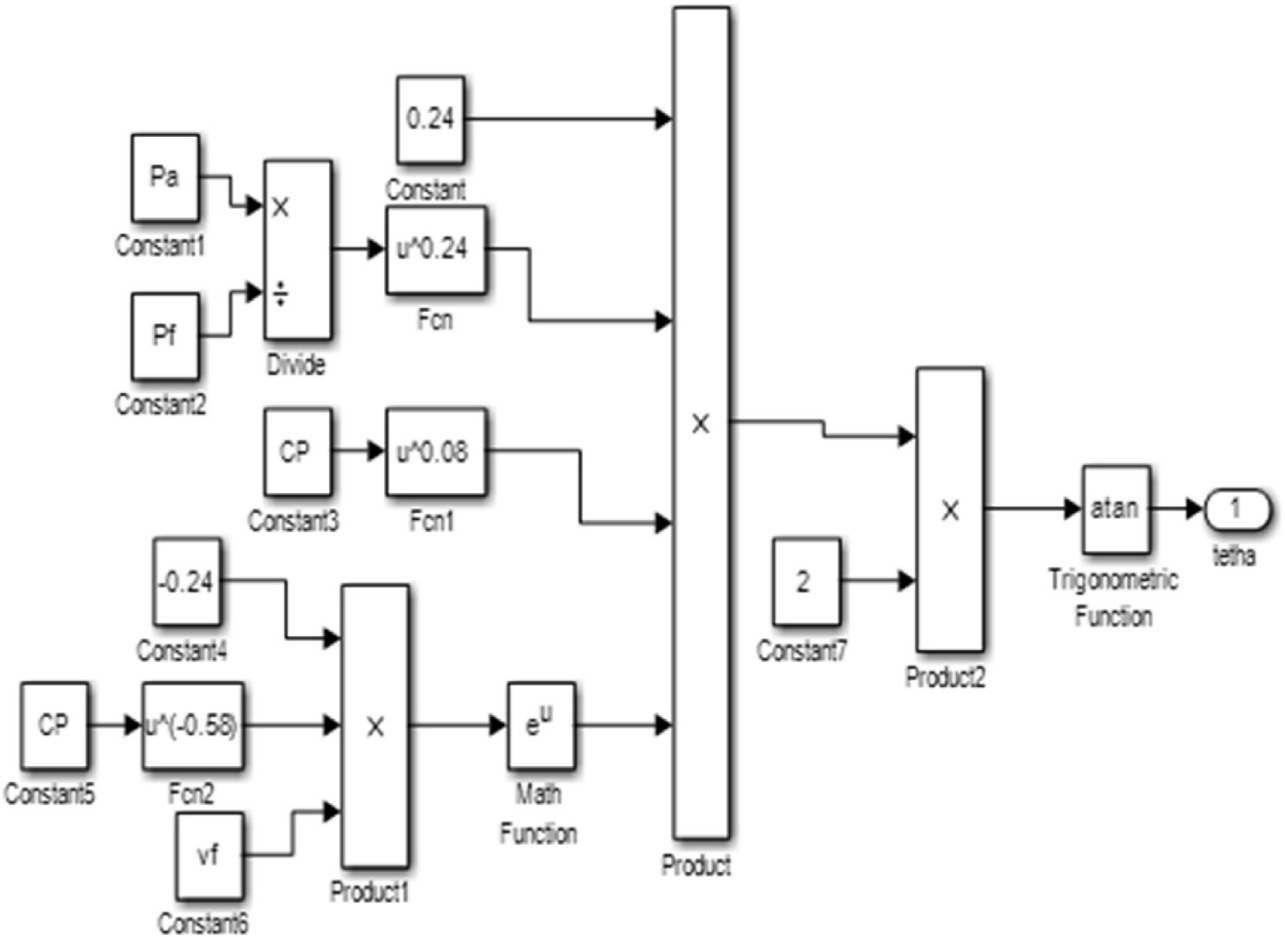
Spray cone angle code block.

Figure 8 gives the code blocks for the determination of the spray cone angle.

#### 2.7.4 Spray area

It is a representation of the quality of the air/fuel mixture. Experimentally, it was determined from the processed image of the spray and then the result thereafter was compared with the numerical result using Eq. (7) as derived here. The spray is taken to be conical in shape. The surface area of a cone is expressed as A=πr(r+s)

From Pythagorean Theorem;

(6) $$S=\sqrt{r^{2}+S{'}^{2}}$$

$r=S.\sin\frac{\theta }{2};S^{'}=S.\cos\frac{\theta }{2}$. Therefore, the spray area is expressed thus:

(7) $$A\left(\text{t}\right)=\pi S{\left(t\right)}^{2}\;\sin\frac{\theta \left(t\right)}{2}+sin^{2}\frac{\theta \left(t\right)}{2}$$

**Figure 9:**
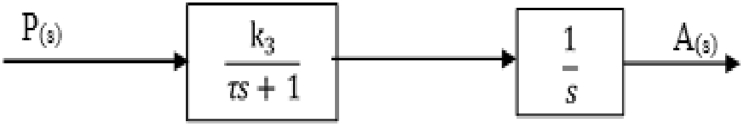
Transfer function block for spray area.

The transfer function block shown in Figure 9 was generated from Eq. (7) and was used to develop code blocks in MATLAB which was run in Simulink to validate the spray area result.

**Figure 10:**
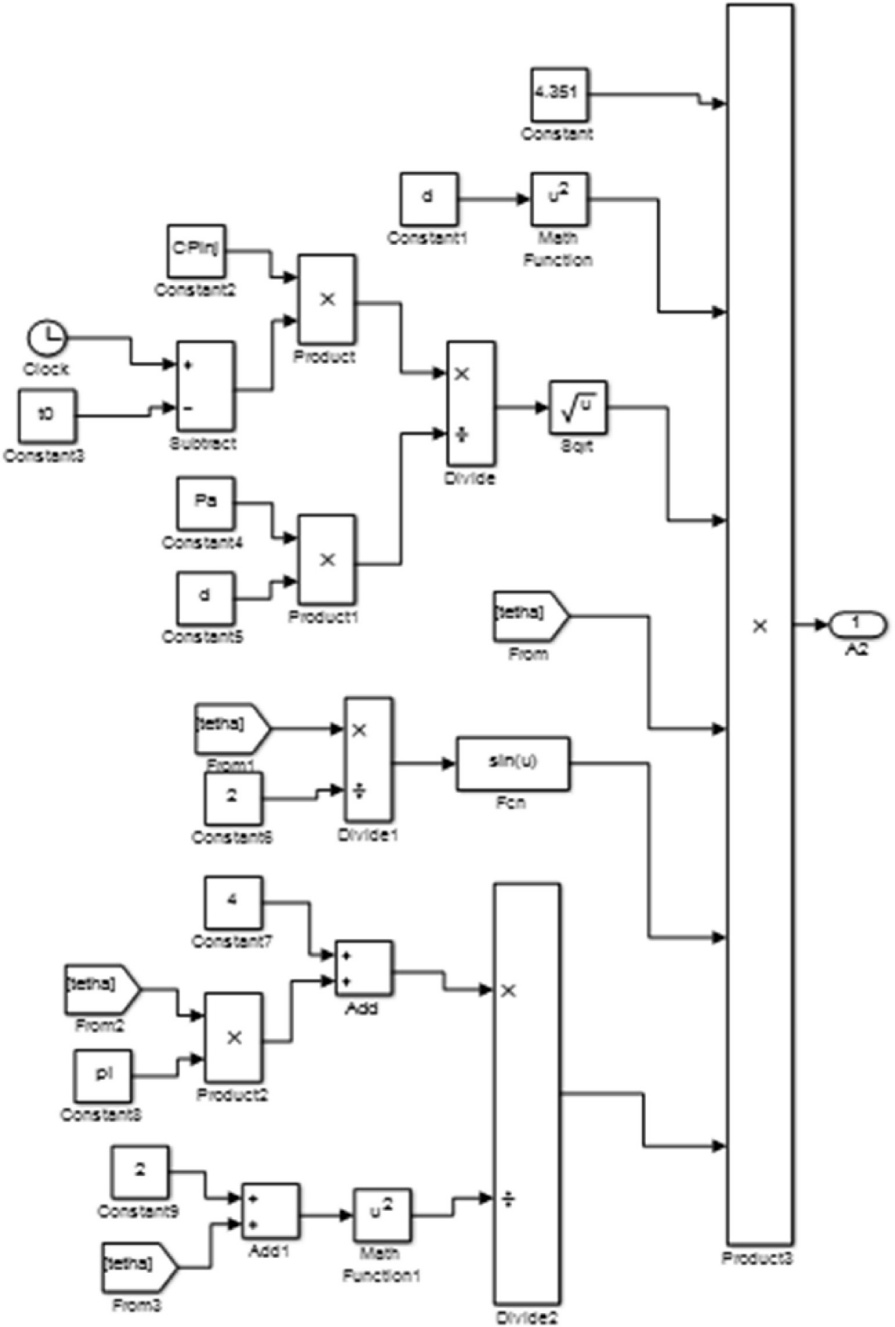
Spray area code block.

Figure 10 gives the code blocks for the determination of the spray area.

#### 2.7.5 Spray volume

The spray volume was evaluated with the following derived expression.

Considering that the volume of a cone, $V=\frac{\pi r^{2}h}{3}$, the spray volume V(t) can be expressed as:

(8) $$\text{V}\left(\text{t}\right)=\frac{\pi S_{\left(t\right)}^{3}}{3}\left(1-cos^{2}\frac{\theta (t)}{2}\right)cos\frac{\theta (t)}{2}$$

S_(t)_ = Spray tip penetration length, and θ(t) = Spray Angle.

**Figure 11:**
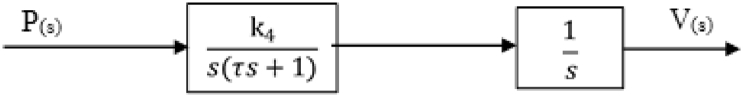
Transfer function block for spray volume.

The transfer function block shown in Figure 11 was generated from Eq. (8) and was used to develop code blocks in MATLAB which was run in Simulink to validate the spray volume result.

**Figure 12:**
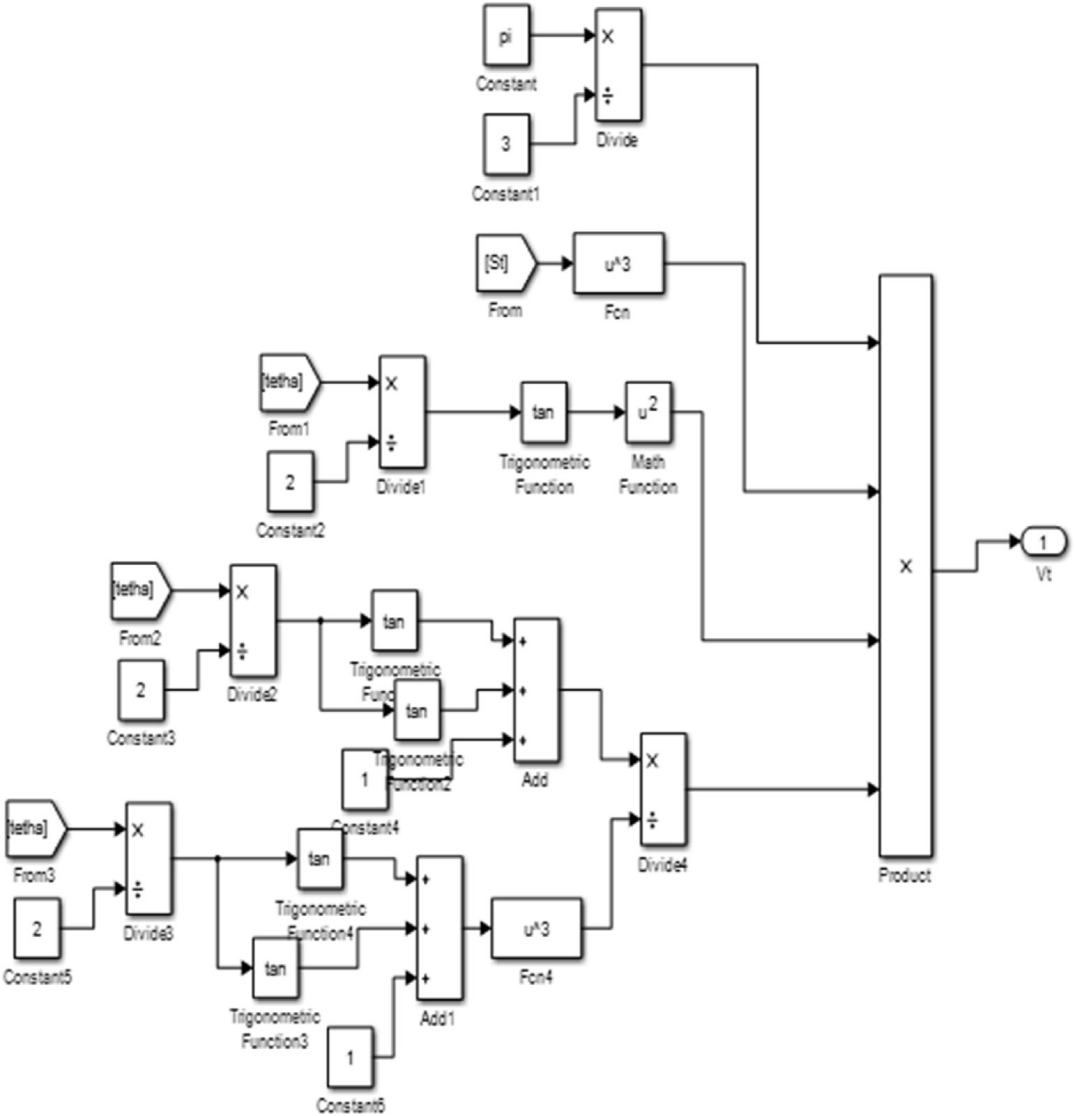
Spray volume code block.

Figure 12 gives the code blocks for the determination of the spray tip velocity.

#### 2.7.6 Sauters Mean Diameter (SMD)

**Figure 13:**
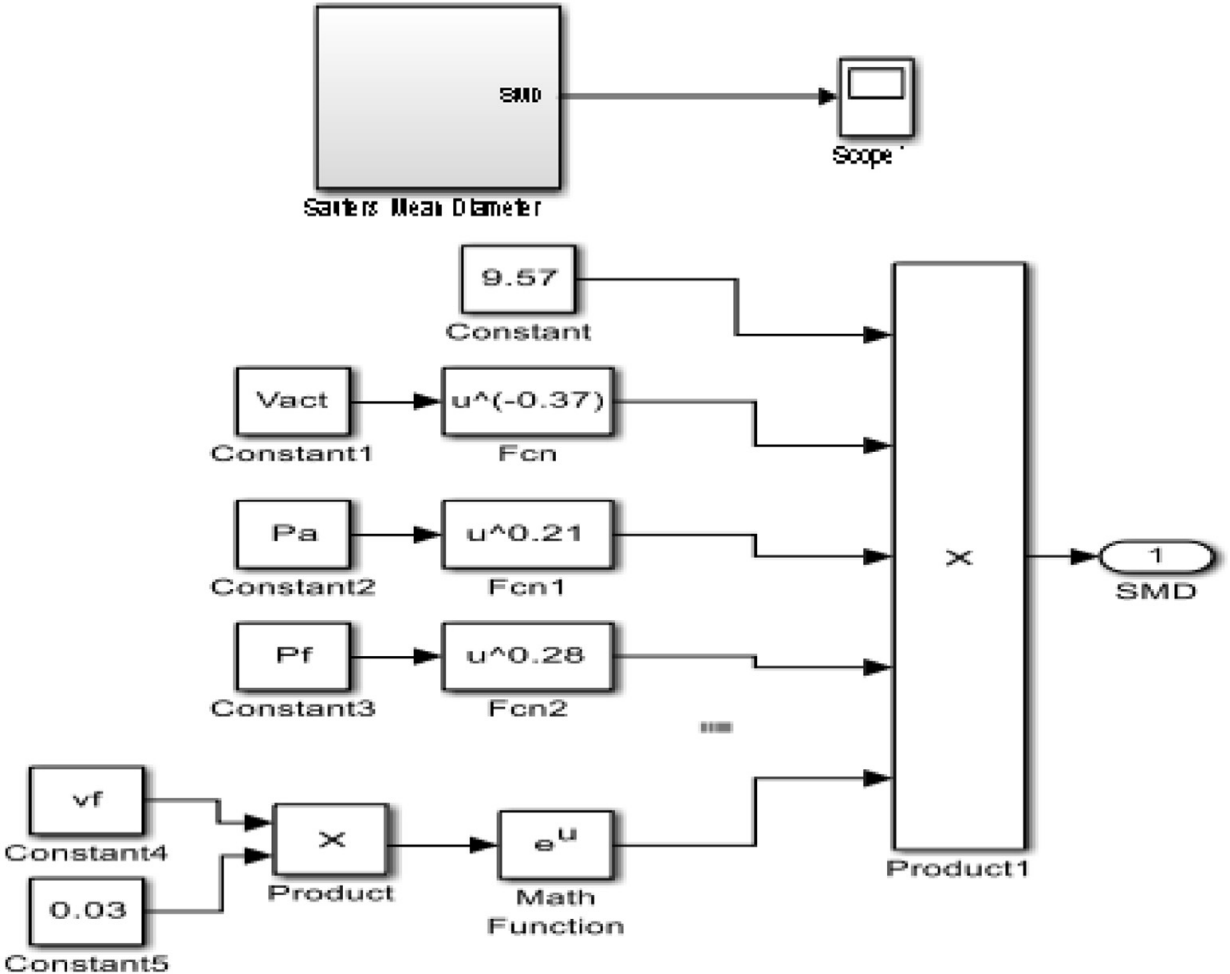
SMD code block.

SMD characterizes fuel atomization and ensures even distribution of the droplets in the injection process hence, determining the combustion range and pollutant exhaust emissions. Due to the inadequacy of the Schlieren technique in determining microscopic spray parameters, the equation established by Dizayi et al [15] was used to generate a Simulink code block in MATLAB as seen in Figure 13 to validate the experimental result.

(9) $$SMD=9.57\times V_{act}^{-0.37}\times \rho_{a}^{0.21}\times \rho_{f}^{0.28}\times e^{0.03vf}$$

Where; $V_{act}=C_{d}V_{th}\;\;;\;\;V_{th}={\left[\frac{2\text{Δ}P}{\rho_{f}}\right]}^{0.5}$, $\rho_{f}$and $\rho_{a}$= density of injected fluid and density of working gas fluid respectively.

## 3 Results and discussion

### 3.1 Spray visualization and processed images

**Figure 14:**
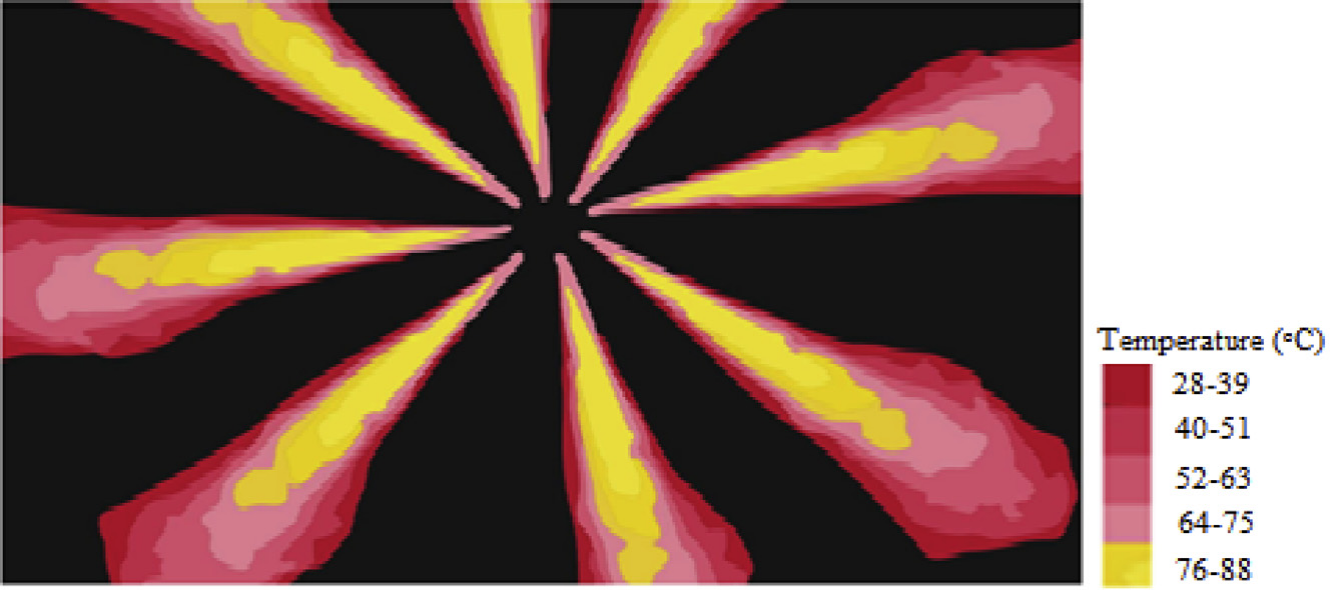
Processed image of spray using image J software for the determination of spray length and spray cone angle.

The results obtained from spray visualization are presented here, and the processed image obtained from the Schlieren visualization set up is shown in Figure 14.

### 3.2 Characterization of oil

The RFO was characterized to determine the physicochemical and thermal properties of the oil, and the result is presented in Table 1.

**Table 1:** Specifications of test fuel.

Parameter	Value
Viscosity @ 28°C (mPa.s)	9.161
Density (kg/m^3^)	867.9
Water content (%)	20.88
Acidity (mg/l)	18.04
Cloud point (°C)	5
Flash point (°C)	69.48
Smoke point (°C)	74.30
Fire point (°C)	94.65
Boiling point (°C)	209.86
Specific heat capacity (kJ/kg°C)	1.88
Cetane number	37
Salinity ppt	0.9735
Pour point (°C)	-4

**Figure 15:**
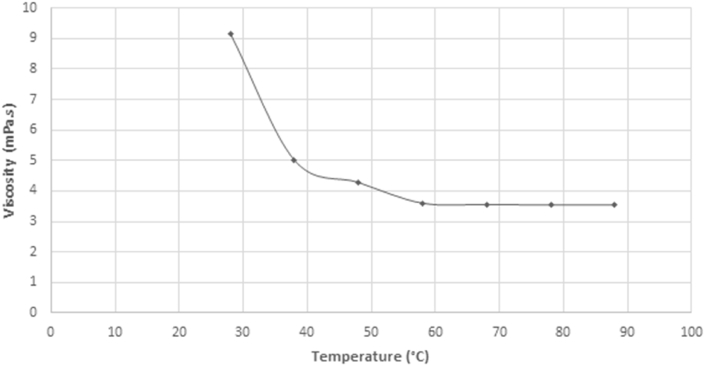
Variation of viscosity against temperature.

Also, to investigate the effect the physical property of viscosity has on the spray parameters, the fuel was subjected to various heating temperatures until a stable value of viscosity was attained. The varying temperature and its corresponding viscosity value are presented in Figure 15.

### 3.3 Spray tip penetration length

An experiment was carried out to determine the effect density and viscosity have on the spray length against various injection times. The experimental result shows that the spray tip penetration length of the fuel increases with increase in time of injection. In order to determine the effect of the physical properties of density and viscosity on the test fuel, it was heated; its density and viscosity values were taken at a temperature interval of 10 °C until a stable viscosity value was obtained. It was observed that at a temperature of 88 °C, the fuel achieved a stable viscosity value as presented in Figure 15.

**Figure 16:**
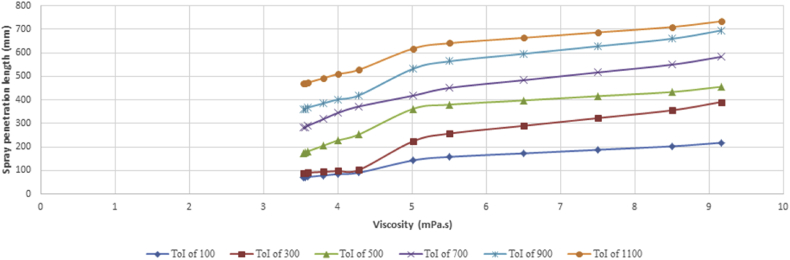
Variation of spray penetration length against viscosity.

However, the density was observed to remain constant during the heating process. This could likely be because the heat supplied by the heat source is utilized to weaken the cohesive forces existing between the molecules of the fuel, leading to no significant change in mass or volume of the fuel, and subsequently no change in the density. Consequently, the study investigated the influence of changes in viscosity on the fuel spray parameters. Figure 16 reveals the effect of viscosity on the spray penetration length of the fuel. It could be seen that changes in the spray penetration length are proportional to changes in viscosity. In other words, a reduction in the viscosity of the fuel leads to a reduction in the spray penetration length.

**Figure 17:**
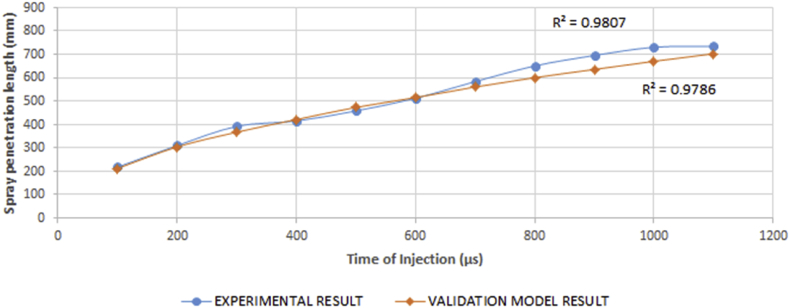
Comparison between experimental and validation model results for variation in spray penetration length against time of injection.

This is due to the weakening of the cohesive forces of the fuel as viscosity reduces. Therefore, at higher viscosity values, longer spray lengths are expected which will lead to splashes on the cylinder walls and subsequently poor combustion. This invariably means the engine will be running on a rich fuel mix after start-off conditions. Figure 16 reveals that the highest and lowest penetration lengths were experienced at the highest and lowest injection times respectively. This phenomenon occurred at a density of 867.9 kg/m^3^ and temperature of 28 °C. Result of the validation model employed agrees with the experimental result as seen in Figure 17. The ANOVA result has a p-value of 0.844. This implies that there is no significant difference between the means of both the experimental and model result. The results correlate by a value of 0.9941 hence, the experimental result agreed with the model result.

A regression model was developed that could predict the spray length for specific values of viscosity and injection time variables, and also find the settings for the values of viscosity and injection time variables that correspond to a desired value or range of values for spray penetration length of the fuel. This model is expressed in Eq. (10).

(10) $$S_{\left(t\right)}=-493.7+170.3\mu +0.3702T-10.35\mu^{2}+0.0170\mu \times T$$

Where S_(t)_ = spray length, μ = viscosity, T = time of injection.

The relationship between the spray penetration length, viscosity and time of injection in the model is statistically significant at p < 0.05, and with an R^2^ value of 98.07%. This means that 98.07% of the variations in the spray length can be explained by the regression model.

### 3.4 Spray cone angle

**Figure 18:**
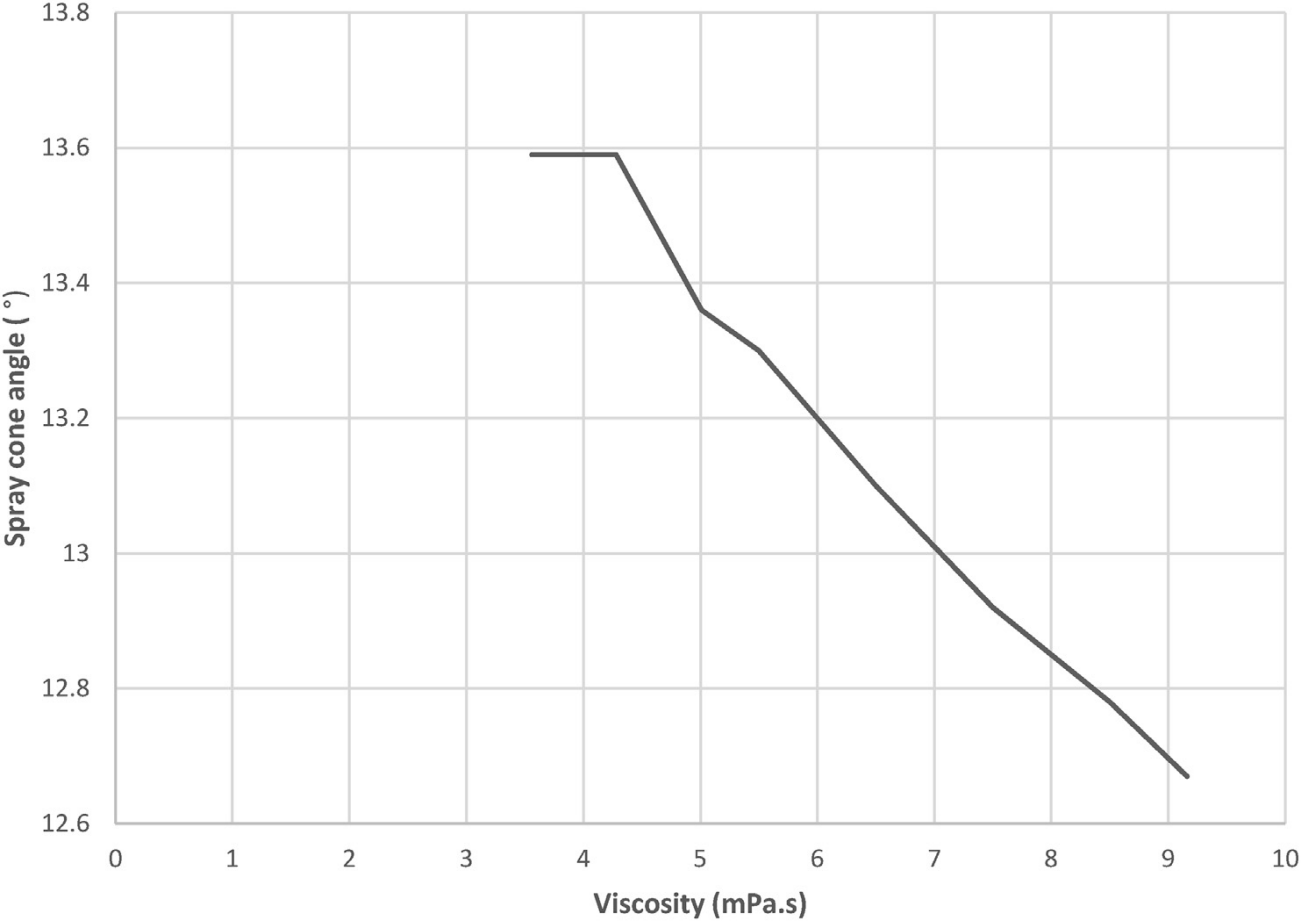
Experimental result for variation in spray cone angle against viscosity.

Result of the spray cone angle reveals that the spray cone angle is independent of the injection time, but is influenced by the viscosity of the fuel. Figure 18 shows that the relationship between the viscosity and spray cone angle is inversely proportional at higher viscosity values and increases monotonically at lower viscosity values.

**Figure 19:**
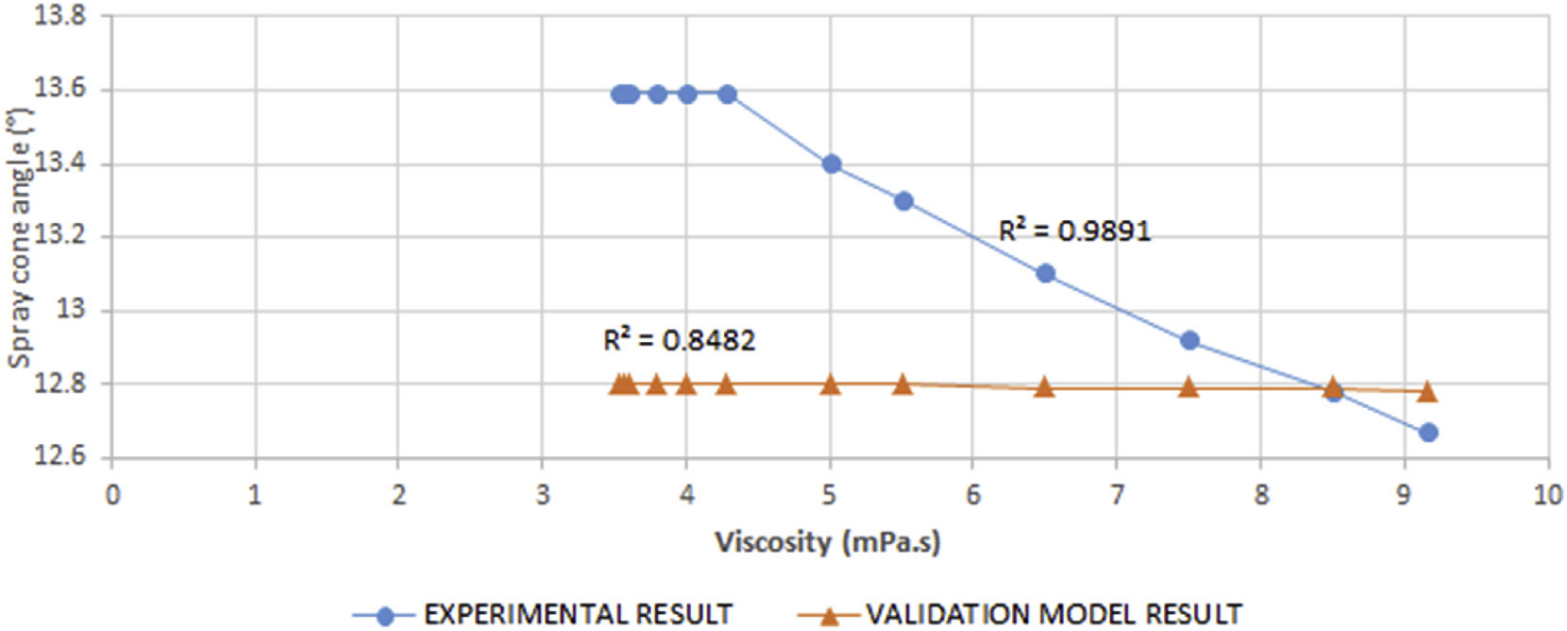
Comparison between experimental and validation model results for variation in spray cone angle against viscosity.

This is so because as the forces of cohesion in the fuel are weakened, the fuel molecules acting under the injected pressure become less localized, and tend to diverge. The ANOVA result correlating the model and experimental values shows that the F value is higher than the F critical value and Figure 19 agrees with the trend observed in the experimental result with a correlation value of 0.9683. The experimental result agreed with the model result due to these indicators. The spray cone angle to a large extent was observed to be instrumental to the spread of the spray in the visualization chamber.

A regression analysis was run on the experimental data to predict values/changes in the spray cone area as a result of changes in time of injection. Result of the regression analysis shows that the R^2^ value is 0.9891, the F_cal_ value is higher than the F_significance_ value and the analysis is statistically significant at p < 0.05. Since F_cal_ > F_significance_ and the p-value is less than the alpha value (level of significance) of 0.05, the regression result is accepted. The regression model is as presented in Eq. (11).

(11) θ=24.344−0.09244μ

The R^2^ value suggests that 98.91% of the variations in the spray cone angle can be explained by the regression model.

### 3.5 Spray area

The spray area was noted to be within very close ranges for the different viscosities examined under any specific intervals of injection time. The spray area was noted to be independent of the viscosity as there was a monotonic change in the spray area with either an increase or decrease in viscosity. However, it was observed that a relationship exists between the spray area and the time of injection. Figure 19 alludes to the proportionality of the relationship that exists between the spray area and the time of injection.

**Figure 20:**
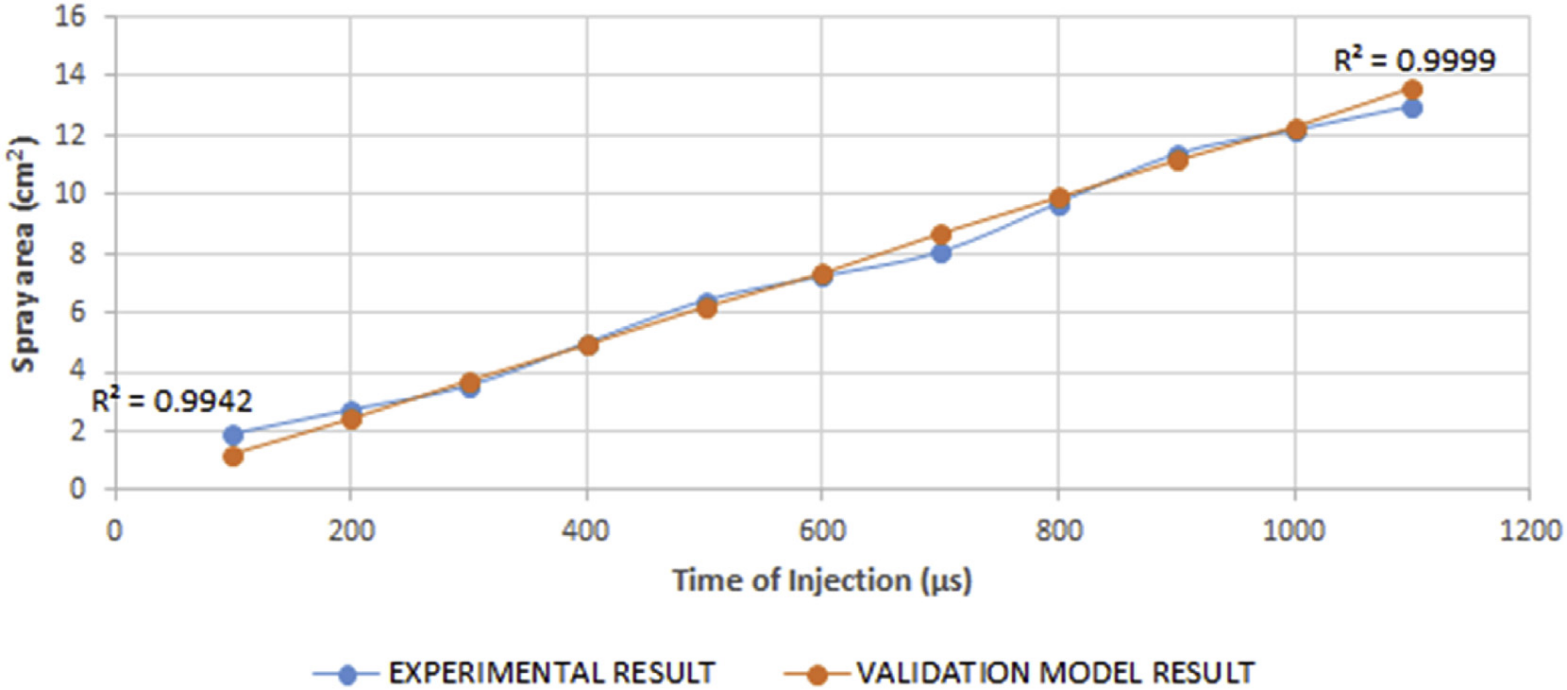
Comparison between experimental and validation model results for variation in spray area against time of injection.

The reason for the monotonic change as regards viscosity is because the spray area is influenced by the spray cone angle. Hence, the monotonic relationship which exists between the spray cone angle and viscosity transcended to the spray area. This implies that conditioning the spray cone angle and area of the fuel within the monotonic regions through heating may prove to be unrealistic. In order to ascertain the veracity of the experimental result, it was compared with the validation model results using ANOVA. The result shows that the P-value is 0.985 which means that the data do not give any reason to conclude that the means of both results differ. The results compared also show that both correlate by a value of 0.9957. This implies that both results are in good agreement with each other hence, the experimental result was accepted. Figure 20 shows that there is an agreement between both results.

A regression model was fit for the trend observed in the spray area of the test fuel. This was done to establish a relationship that could be helpful to determine the appropriate injection time that would be needed for the desired spray area during fuel modification processes. Result of the regression analysis shows that the R^2^ value is 0.994, the F_cal_ value is higher than the F_significance_ value and that the analysis is statistically significant at p < 0.05. Since F_cal_ >> F_significance_ and the p-value is less than the alpha value (level of significance) of 0.05, the regression result is accepted. The regression model is shown in Eq. (12).

(12) Á=0.485+0.01149T

The relationship between the spray area and time of injection in the model is statistically significant at p < 0.05, and 99.4% of the variations in the spray area can be explained by the regression model.

### 3.6 Spray volume

**Figure 21:**
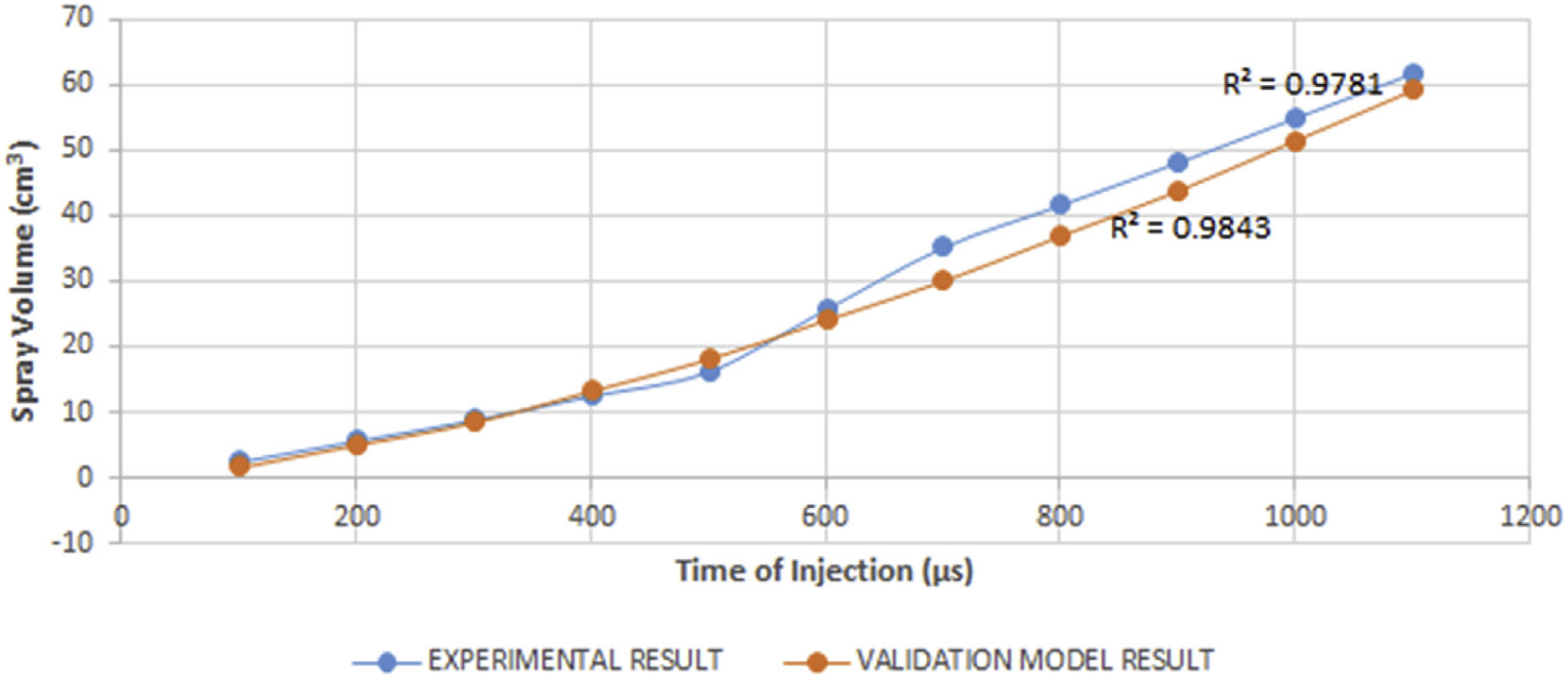
Comparison between experimental and validation model results for variation in spray volume against time of injection.

The viscosity did not record a significant change on the spray volume across its values when compared with specific injection time. The spray volume was not affected by changes in the fuel viscosity rather it was influenced by the time of injection. The spray volume increases with an increase in the injection time. Figure 21shows that the relationship between the spray volume and the injection time is proportional. This phenomenon is expected as the time of injection indicates how long the injector nozzle valve will stay open. Therefore, the longer the open condition duration, the more the volume discharged. Also, the non-dependence of the spray volume on the viscosity of the fuel is owing to the monotonic relationship that exists between the spray cone angle and the viscosity.

The correctness of the experimental result was compared with the model result using ANOVA. The ANOVA for the model and experimental results shows that p-value of 0.888 suggests that there is no significant variation from the mean of both results. With a correlation value of 0.9956, both results agree with the proportional relationship that exists between the spray volume and the time of injection, hence, the experimental result is accepted. Figure 21 gives a graphical representation of the agreement.

A regression analysis was carried out on the experimental result to develop a model that could predict the spray volume for specific values of injection time, and also find the settings for the values of injection time variables that correspond to a desired range of values for spray volume of the fuel. Result of the analysis show that the R^2^ value is 0.9781, the F_cal_ value is higher than the F_significance_ value, and the analysis is statistically significant at p < 0.05. Since F_cal_ >> F_significance_ and the p-value is less than the alpha value (level of significance) of 0.05, the regression result is accepted. This model is expressed in Eq. (13).

(13) V=−8.358+0.06178T

The relationship between the spray volume and time of injection in the model is statistically significant at p < 0.05, and 97.81% of the variations in the spray area can be explained by the regression model.

### 3.7 Spray tip velocity

**Figure 22:**
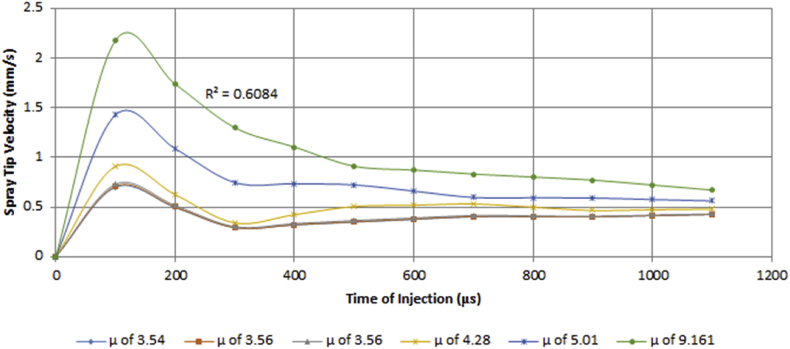
Variation of spray velocity against time of injection for various values of viscosity.

The spray velocity was noticed to depend on both the time of injection and viscosity. The experimental result revealed that an inverse proportional relationship exists between the spray velocity and the time of injection at early spray stages. As the spray progresses, the frictional drag of the liquid droplets leads to a loss in its kinetic energy which translates to less energetic molecules and subsequently less spray velocity. Figure 22 shows that there is a decrease in the spray velocity for all the various viscosity values as the injection time increases.

**Figure 23:**
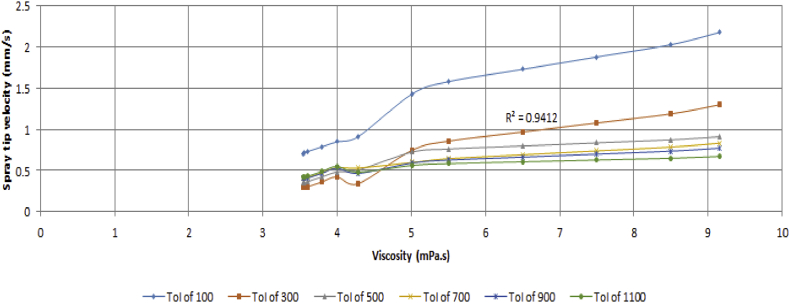
Variation of spray velocity against viscosity for various values of time of injection.

The spray velocity was noted to be directly proportional to the change in viscosity of the test fuel. Therefore, an increase or decrease in the viscosity will lead to a corresponding increase or decrease in the spray velocity. Figure 23 portrays the variation in the spray tip velocity with viscosity.

A regression model was fitted for the trend observed among the spray velocity, viscosity and time of injection of the test fuel. This was done to establish a relationship that could be helpful to determine the appropriate injection time and viscosity of the fuel that would be needed for the desired spray velocity. The result from the regression analysis shows that the R^2^ value is 0.6084, the F_cal_ value is higher than the F_significance_ value and the analysis is statistically significant at p < 0.05. Since F_cal_ > F_significance_ and the p-value is less than the alpha value (level of significance) of 0.05, the regression result is accepted. This expression is shown in Eq. (14).

(14) $$U=-0.821+0.546\mu -0.000887T-0.2329\mu^{2}+0.00000122T^{2}-0.0002118\mu T$$

The relationship between the spray velocity, viscosity and time of injection in the model is statistically significant at p < 0.05, and 60.84% of the variations in the spray velocity can be explained by the regression model.

### 3.8 Sauter Mean Diameter

The SMD of the fuel is a microscopic parameter which cannot be determined by the visualization apparatus because the visualization apparatus uses conventional photographic techniques which do not investigate the spray within the region of the nozzle tip where the spray is dense. Hence the Simulink code blocks as shown in Figure 13 were used to determine the effect viscosity has on the SMD of the fuel. The SMD of the fuel was constant for the various values of viscosity. It was observed that the SMD is independent of the viscosity, but depends on the injection pressure and the density of the fuel. The SMD gives the atomization capacity of the fuel; the lower the percentage the better the atomization of the fuel. The fuel had an atomization value of 2.85mm.

### 3.9 Comparison between the RFO and diesel fuel spray parameters

From the spray parameters examined, the spray tip penetration length, spray cone angle and SMD chiefly influence other parameters and as a result, determine the spray pattern of the injected fuel. The spray length influences the velocity profile of the spray while the spray cone angle influences the area and volume of the spray while the SMD influences the atomization of the fuel. Therefore, in comparing the spray parameters of RFO with diesel fuel, attention is given to the spray tip penetration length and the spray cone angle. Table 2 gives a comparison of both fuels under the same operating conditions.

**Table 2:** Fuel physical properties and spray parameter comparison.

Fuel Type	Residual Fuel Oil	Diesel fuel
**Physical properties**		

Density @ 28 °C	867.9 kg/m^3^	832 kg/m^3^
Viscosity @ 28 °C	9.161 mPa.s	3.35 mPa.s

**Fuel spray conditions**		

Injection pressure	40 MPa	40 MPa
Ambient pressure	0.1 MPa	0.1 MPa
Time of Injection	500 μs	500 μs

**Spray parameter**		

Spray length	456 mm	256 mm [12, 18]
Spray cone angle	12.69°	17°–23° [16, 17, 19]
Sauter Mean Diameter	2.85 mm	0.715mm [20]

From the result displayed in Table 2, it could be seen that there is a disparity between spray length, spray cone, and SMD values of both fuels. The SMD is seen to be four times larger than that of diesel fuel. This implies that the molecule sizes of the RFO are four times larger than that of diesel under the same operating condition and therefore, poor atomization is expected. This will eventually affect the combustion quality of the fuel. Also, the high spray length and low spray cone angle values of RFO will lead to a higher spray volume causing the spray to impinge on the walls of the cylinder. As a result of the excess fuel present, the engine continues to run on a rich mixture after initial start-up conditions. Attendant consequences of the rich mixture experienced include a reduction in power developed and an increase in carbon deposit. The accumulated carbon deposit as a result of incomplete combustion poses a nuisance to the injector by clogging the nozzle when the generator is shut down. This makes spray difficult or sometimes impossible during a restart of the generator.

**Figure 24:**
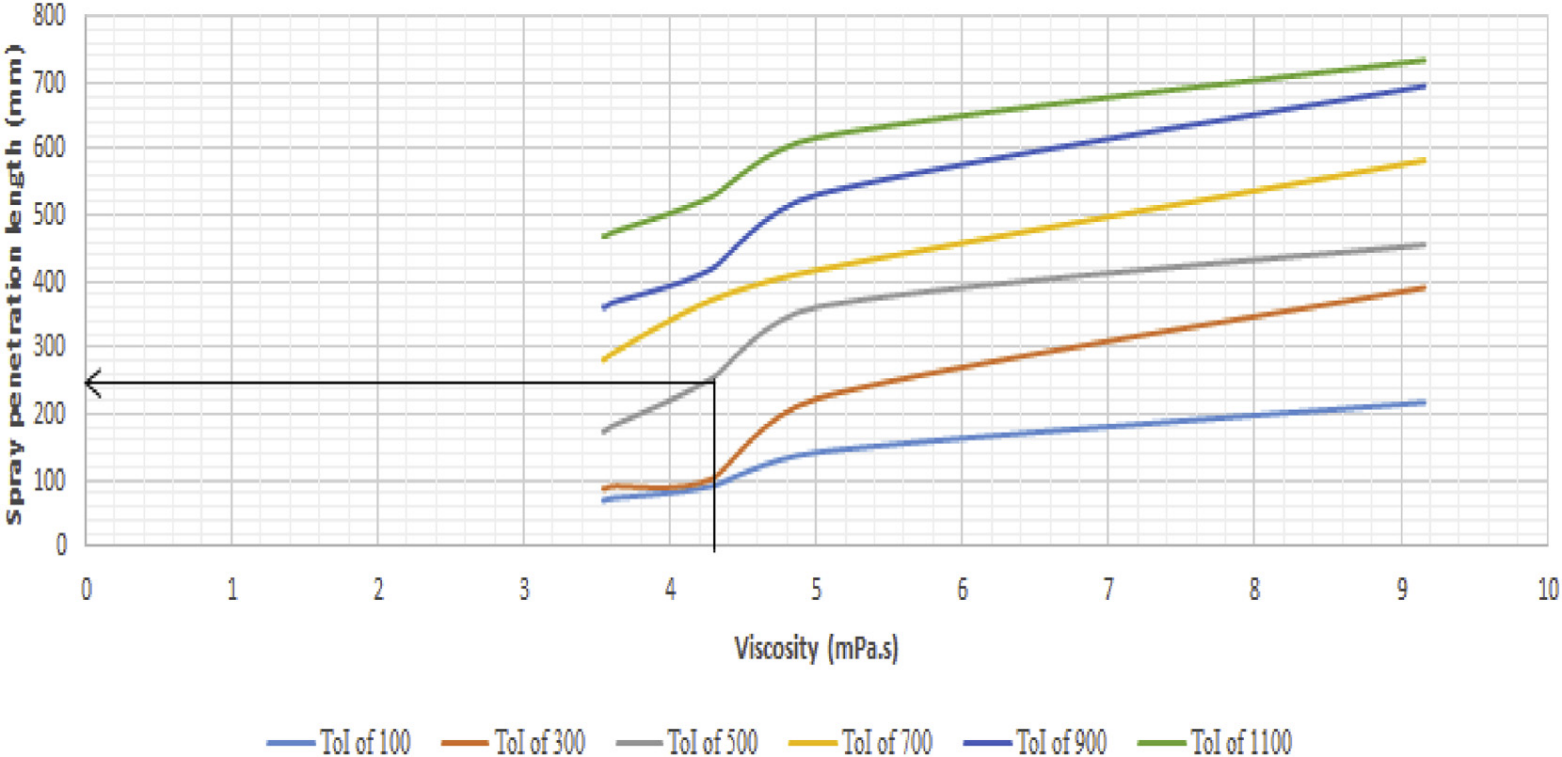
Spray length versus viscosity chart of residual fuel oil at 867.9 kg/m^3^.

Hence, to resolve this issue, the fuel can be conditioned to possess a viscosity that would allow for a spray parameter within close range of that of the diesel fuel. To achieve this, the regression model expressed in Eq. (10) was employed, and it was discovered that at viscosity of 4.305 mPa.s, the spray length would be approximately 256mm. This finding is roughly supported by the plot of spray length against viscosity chart of the fuel as seen in Figure 24.

**Figure 25:**
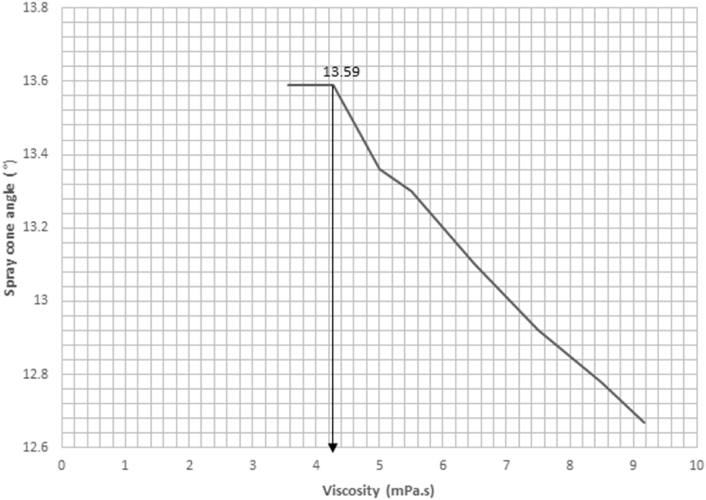
Spray cone angle versus viscosity chart of residual fuel oil at 867.9 kg/m^3^.

From the spray cone angle versus viscosity chart, it is seen that at the viscosity value of 4.305 mPa.s, the spray cone angle will expand to the monotonic region. This increase in angle as the spray shortens will lead to a better spread of the fuel within the combustion chamber. Figure 25 shows the change in cone angle from a spray cone angle versus viscosity chart.

**Figure 26:**
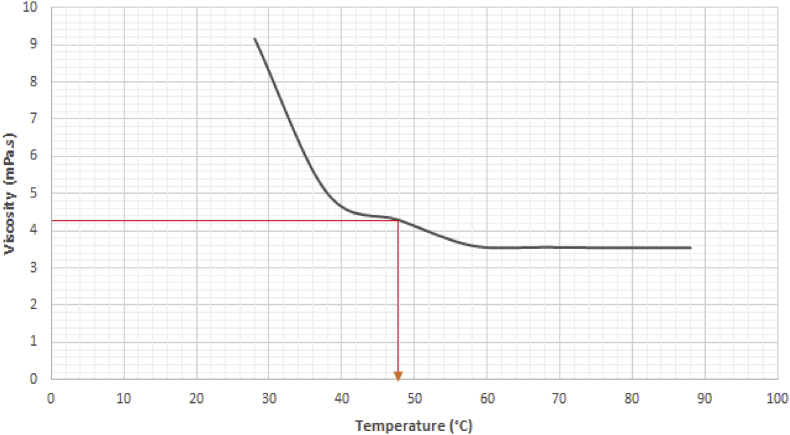
Viscosity versus temperature chart of residual fuel oil at 867.9 kg/m^3^.

To achieve this viscosity value, the viscosity versus temperature chart of the fuel is used to determine the temperature range at which this viscosity is attainable. Figure 26 shows that when the fuel is conditioned to a working temperature of 48 °C, a viscosity of 4.305 mPa.s can be obtained.

To maintain a temperature of 48 °C heating of the oil has to be in two stages. The heating time needed to raise the temperature of the fuel to 48 °C in the first heating stage is expressed in Eq. (15).

(15) $$t=\frac{\rho VC_{f}\left(48{}^{\circ}\text{C}-\theta_{i}\right)}{P}$$

Where: ρ=fuel density, V= volume of reservoir, $C_{f}$= specific heat capacity of fuel, $\theta_{i}=$initial temperature of the fuel, P= power rating of the heating element.

The second heating process is done following the fuel consumption rate of the generator. The second fuel tank acts as an external fuel tank for the generator, and as such, the fuel working temperature of 48 °C should be maintained. The electric power needed to maintain a temperature of 48 °C is given in Eq. (16).

(16) $$P=\rho {\dot{V}}_{f}C_{f}$$

Where: ${\dot{V}}_{f}=fuel\;consumption\;rate=\frac{{\dot{m}}_{f}x3.6x10^{6}}{\rho }\left(l/hr\right)$

## 4 Conclusion

The following conclusions were drawn from the investigation; the spray tip penetration length of both fuels increases with increase in time of injection, the viscosity of RFO is higher than the viscosity of conventional diesel, and RFO has a longer penetration length than diesel, also changes in the spray penetration length of both fuels is proportional to changes in viscosity. Diesel fuel has a smaller spray cone angle than RFO, and the relationship between the viscosity and spray cone angle is inversely proportional at higher viscosity values and increases monotonically at lower viscosity values for both fuels. The spray area is independent of viscosity as there exists a monotonic change in the spray area with either an increase or decrease in viscosity. However, a proportional relationship exists between the spray area and the time of injection. The spray volume for both fuels is not affected by changes in the fuel viscosity rather it is influenced by the time of injection. The spray volume increases with an increase in the injection time. The spray velocity depends on both the time of injection and viscosity. From the experimental result, an inverse proportional relationship exists between the spray velocity and the time of injection at early spray stages. As the spray progresses, the frictional drag of the liquid droplets leads to a loss in its kinetic energy which translates to less energetic molecules and subsequently less spray velocity. Spray parameter values of RFO are higher than those of diesel fuel. The higher spray parameter values of RFO leads to a higher spray volume causing the spray to impinge on the walls of the cylinder. As a result of the excess fuel present, the engine continues to run on a rich mixture after initial start-up conditions. Attendant consequences of the rich fuel mixture experienced include a reduction in power developed, and an increase in carbon deposit. The accumulated carbon deposit as a result of incomplete combustion poses a nuisance to the injector by clogging the nozzle when the generator is shut down. This makes spray difficult or sometimes impossible during a restart of the generator. Challenges associated with the usage of RFO can be eliminated by conditioning the fuel to possess a viscosity that would allow for a spray parameter within close range of that of the diesel counterpart. This viscosity was determined to be 4.305 mPa.s, for a spray length of 256mm when the engine operates at an injection time of 500μs.

## Declarations

### Author contribution statement

Achebe C.H.: Conceived and designed the experiments; Wrote the paper.

Ogunedo B.M.O.: Conceived and designed the experiments; Analyzed and interpreted the data; Contributed reagents, materials, analysis tools or data.

Chukwuneke J.L.: Performed the experiments; Analyzed and interpreted the data; Wrote the paper.

Anosike N.B.: Performed the experiments; Contributed reagents, materials, analysis tools or data.

### Funding statement

This research did not receive any specific grant from funding agencies in the public, commercial, or not-for-profit sectors.

### Competing interest statement

The authors declare no conflict of interest.

### Additional information

No additional information is available for this paper.
